# Clinical outcomes following periodontal surgery and root surface decontamination by erythritol-based air polishing. A randomized, controlled, clinical pilot study

**DOI:** 10.1007/s00784-020-03533-9

**Published:** 2020-08-24

**Authors:** Raluca Cosgarea, Søren Jepsen, Rolf Fimmers, Aura Bodea, Sigrun Eick, Anton Sculean

**Affiliations:** 1 Department of Periodontology, Operative and Preventive Dentistry, University of Bonn, Welschnonnenstr. 17, 53111 Bonn, Germany; 2 Department of Prosthetic Dentistry, University Iuliu-Hatieganu, Str. Clinicilor nr 32, 400006 Cluj-Napoca, Romania; 3 Department of Periodontology and Peri-implant Diseases, Philipps University of Marburg, Georg-Voigt Str. 3, 35033 Marburg, Germany; 4 Institute for Medical Biometry, Informatics and Epidemiology, University Bonn, Venusberg-Campus 1, 53127 Bonn, Germany; 5 Periodontal Private Practice for Periodontology, Gheorghe Doja Str. 9, 400068 Cluj-Napoca, Romania; 6 Department of Periodontology, University Bern, Bern, Switzerland, Freiburgstrasse 7, CH-3010 Bern, Switzerland

**Keywords:** Surgical periodontal treatment, Air polishing, Supra-alveolar defects, Erythritol, Root surface decontamination

## Abstract

**Aim:**

To evaluate the outcomes following surgical periodontal treatment and root surface decontamination by means of air polishing using an erythritol powder or conventional mechanical root debridement.

**Material and methods:**

Thirty systemically healthy patients (44.38 ± 8.2 years old, 11 smokers, 19 women) diagnosed with periodontitis stages III–IV were included. Each patient, with one single-rooted tooth, with one probing pocket depth (PD) ≥ 6 mm associated with horizontal bone loss, was treated by means of simplified papilla preservation flap (SPPF) and randomized to either test treatment (careful removal of the calculus with the tip of a blade, air polishing of the root surfaces with erythritol) or to the control group (scaling and root planing with hand curettes, ultrasonic instruments). PD, clinical attachment (CAL), bone sounding (BS), and radiographic bone level (BL) were evaluated at baseline and 12 months postsurgically.

**Results:**

Twenty-seven patients completed the 12-month follow-up (test: *n* = 14, control: *n* = 13). In both groups, statistically significant improvements were obtained (*p* < 0.05, mean CAL gain/PD reduction: test, 2.50 ± 1.60 mm/3.00 ± 0.96 mm; control, 2.85 ± 1.21 mm/3.38 ± 1.12 mm). No statistically significant differences were observed between the groups for any of the investigated parameters (*p* < 0.05).

**Conclusion:**

Within their limits, the present results indicate that the use of air polishing with an erythritol powder during periodontal surgery may represent a valuable minimally invasive adjunct following calculus removal by means of hand and ultrasonic instruments or a valuable alternative to these, for root surfaces without calculus.

**Clinical relevance:**

The use of air polishing with an erythritol powder during periodontal surgery appears to represent a valuable minimally invasive adjunct following calculus removal by means of hand and ultrasonic instruments or a valuable alternative to these, for root surfaces without calculus.

## Introduction

The main goal of periodontal therapy is to arrest further attachment loss and, consequently, prevent further disease progression and subsequent tooth loss. In most cases, this goal can be predictably achieved by means of non-surgical periodontal therapy using hand and ultrasonic instruments with or without antibiotics [1]. However, in certain cases, residual pockets may still persist. Long-term clinical studies have demonstrated that residual pockets ≥ 6 mm and bleeding on probing (BOP) represent a risk for further increase of the pocket depth and loss of clinical attachment (CAL). Moreover, teeth exhibiting residual pockets with probing depths (PD) ≥ 6 mm and BOP were at higher risk for extraction/tooth loss on long-term basis [2]. Consequently, such sites are indicated for additional corrective surgical therapy aiming to decrease probing depths or/and to reconstruct the bony defects [3, 4].

However, the extent of root surface debridement during corrective periodontal surgery is still a matter of debate. While some authors recommend complete removal of calculus, plaque and “diseased cementum” [5], others support a “less aggressive” root surface decontamination for example through polishing [6–10]. Results from preclinical and clinical studies appear also to support a “less aggressive” approach since comparable histological results in dogs and clinical improvements in humans were obtained following periodontal surgery with either complete removal of the “diseased” root cementum or following root surface polishing only [6–8]. Thus, it can be anticipated that, since the loosely adhering subgingival dental plaque can also be removed by gentle scaling or chemical conditioning of the root surface, the intentional removal of the entire cementum layer may not be necessary in order to improve clinical outcomes, since the loosely adhering subgingival dental plaque can also be removed by gentle scaling or chemical conditioning of the root surface [11–13].

Furthermore, from a clinical point of view, it is important to point out that extensive scaling and root planing often results in an additional increase of root hypersensitivity, thus impairing the patient’s quality of life [14]. Taken together, these data suggest that root surface instrumentation should be minimally invasive, primarily focussing on calculus removal and effective disruption/removal of the biofilm rather than on excessive and intentional removal of root cementum [15, 16].

Only few air-abrasive materials and methods have thus far been investigated for subgingival plaque removal. One of these methods is an air-polishing device using a low-abrasive glycine powder (GPAP) which seems to be an effective alternative for removing the subgingival biofilm. GPAP was shown to result in greater reductions of colony-forming bacterial units in moderate pockets (3–5 mm PD) compared with hand instruments [9]. Additionally, the abrasiveness of GPAP was found to be approximately 80% lower than air polishing with a bicarbonate powder and resulted in less loss of hard tissue as compared with the use of hand or sonic instruments [17]. Moreover, it has been also shown that GPAP was safe and caused statistically significantly less pain by taking less time compared with conventional instrumentation. The short-term microbiological effects (i.e. at 7 days) were also comparable with those obtained following conventional instrumentation [18, 19].

Another investigated material for subgingival biofilm removal is erythritol. Erythritol is a polyol, an artificial non-cariogenic sweetener, worldwide accepted as a food additive, proven to be non-toxic, chemically neutral and highly water-soluble [20, 21]. In vitro investigations have shown that the abrasiveness and particle size of erythritol are comparable with that of glycine [22]. A more recent in vitro study provided evidence that air polishing with erythritol or erythritol plus chlorhexidine assures a better biofilm removal with less substance loss compared with manual instrumentation leading to a smooth surface with nearly no residual biofilm, promoting the reattachment of PDL-fibroblasts [23].

Results from a randomized controlled clinical trial (RCT) showed comparable clinical outcomes at 3 and 6 months after scaling and root planing (SRP) or the subgingival appliance of an air-polishing device with erythritol (EPAP) for patients in supportive periodontal therapy (SPT) [22, 23]. However, a statistically significantly better acceptance was obtained among subjects receiving EPAP compared with conventional SRP. Thus, the available data suggest that the used erythritol powder applied subgingivally with an air-polishing device is well tolerated and safe and can be considered for repeated instrumentation of residual pockets during SPT [22].

Based on the mentioned data, the question arises whether root surface decontamination using air polishing may also represent a valuable modality for effective biofilm removal and root surface decontamination during periodontal surgery.

Therefore, the aim of this pilot study was to determine the healing following treatment of supra-alveolar bony defects by means of conventional periodontal surgery (e.g. access flap using a simplified papilla preservation flap, SPPF) using an erythritol powder applied with an air-polishing device in relation to the use of conventional hand and ultrasonic instruments.

## Material and methods

### Patients and study design

This was a single-centre, examiner-masked, two-arm parallel design randomized controlled pilot study that was conducted according to the Declaration of Helsinki (1964, revision 2008) and approved by the University of Medicine and Pharmacy Cluj-Napoca Ethical Committee (Application #201/25.19.2013). The study was registered in the ISRCTN registry (ISRCTN41294401, http://www.isrctn.com/ISRCTN41294401). The study was planned and conducted as a feasibility study in order to assess the healing including clinical outcomes and side effects of root surface decontamination by means of an erythritol powder and air-polishing device in conjunction with periodontal surgery, as related to the standard therapy of open flap debridement using conventional hand and ultrasonic instruments for root surface decontamination.

Thirty systemically healthy patients (no infectious/heart diseases with need of prophylactic administration of antibiotics before dental treatments, no down syndrome, HIV, diabetes mellitus types I and II, no liver diseases) with periodontitis stages III and IV were included in the study.

In order to be included, patients had to present the following inclusion criteria:

- One single-rooted tooth with a PD ≥ 6 mm and horizontal bone loss with a maximum 2-mm intrabony component as detected radiographically and later confirmed clinically during surgery (experimental tooth)
- Over 18 years old
- To have completed the phase of non-surgical periodontal therapy (initial anti-infective therapy) at least 3 months prior to study inclusion or be in the corrective phase of the periodontal treatment or engaged in SPT
- To maintain a good level of oral hygiene (plaque control record (PCR) after O’Leary 1972 ≤ 25%] [24]

Exclusion criteria were as follows:

- Periodontal surgery at the experimental teeth in the past 12 months
- Test teeth with clinical and/or radiographic signs of a vertical/horizontal tooth fracture or occlusal trauma
- Pregnancy or breastfeeding
- Patients smoking > 10 cigarettes per day [25]

Informed written consent to participate in this study was obtained from all participants prior to study commencement.

### Clinical protocol

All included patients had one single experimental tooth with one test site (PD ≥ 6 mm) treated within this study. Experimental teeth were considered single-rooted teeth with at least one site with PD ≥ 6 mm (test site) and radiographic evidence of horizontal bone loss/vertical bone loss ≤ 2 mm. Teeth with mobility grade II or higher [26] were not included.

The examiner (A.B.) was calibrated prior to study commencement by measuring PD, CAL and mobility in five patients with minimum of 10 teeth twice 48 h apart (mean intra-examiner reliability: PD, 0.87; CAL, 0.76, Cohen’s Kappa Analyses).

Following parameters were recorded by the same, masked to the treatments, and calibrated periodontist (A.B.) at baseline (prior to surgery): PD, clinical vertical attachment level (CAL) at 6 sites per tooth with a mm-scaled periodontal probe (PCPUNC 15; Hu Friedy®, Chicago, IL, USA), bone sounding (BS) at the nearest 0.5 mm and mobility [26]. As a reference point for CAL and BS measurements, the cemento-enamel junction (CEJ) was used. If the CEJ was covered by a restoration (filling/crown), the margin of the restoration was taken as a reference point. Additionally, bleeding on probing (BOP) and full-mouth plaque scores (FMPS) [24] were also recorded. Additionally, periapical radiographs from the experimental teeth were taken with the long-cone paralleling technique using individual film holders.

All surgical procedures at the experimental teeth were performed under local anaesthesia by the same experienced periodontist (R.C.). In both groups, experimental teeth were accessed using a SPPF [27] as follows: intracrevicular incisions were performed at the experimental site (PD ≥ 6 mm) of the tooth and its neighbouring tooth, followed by an oblique interdental incision with a 15C blade (Stoma®, Germany); mucoperiosteal flaps were then raised vestibularly and orally; vertical releasing incisions were avoided. All granulation tissue from the inner aspect of the flap and the alveolar bone was removed using Gracey curettes (Hu Friedy®, Chicago, IL, USA) and tissue scissors. No osseous surgery was performed.

According to a computer-generated randomization list (block randomization), patients were chronologically advised the randomization number (RB). Experimental sites were treated as follows:

- Test group: Calculus, only if present, was carefully removed (“chipped off”) with the tip of a blade, in order to avoid or minimize cementum removal from the root surfaces. The root surfaces at the experimental site were decontaminated by means of an erythritol powder air-polishing device applied using a single-use subgingival nozzle (mid water and power settings for 10 s; Air-Flow Master with Perio-Flow System, EMS®, Nyon, Switzerland). The nozzle was kept in contact to the root surfaces, and vertical strokes in corono-apical direction were performed for 10 s [23], thus enabling the erythritol powder to reach the root surface perpendicularly.
- Control group: Conventional SRP of the root surface at the experimental site by means of hand (10 strokes at average working pressure using Gracey curettes, Hu Friedy®, Chicago, IL, USA) and ultrasonic instruments (mid water and power settings for 10 s; Air-Flow Master with Perio-Flow System, EMS®, Nyon, Switzerland).

Subsequently, the flaps were repositioned and sutured using double sling sutures (5.0 Medilene, Stoma®, Germany). Postoperatively, all patients brushed their teeth excepting the operated area with a chlorhexidine digluconate tooth paste (Elugel®, Pierre Fabre, Paris, France) and rinsed with 0.2% chlorhexidine digluconate solution (Corsodyl®, GlaxoSmithKline, Brentford, London, UK) for 2 min twice daily for 1 week until suture removal. Recall appointments were scheduled at 3, 6 and 12 months after surgery and consisted of supragingival professional tooth cleaning and oral hygiene instructions. During the first 6 months, neither subgingival instrumentation nor probing of the operated area was performed. At the 6 and 12 months appointments, all baseline recorded parameters (PD, CAL, BS, GBI, BOP, FMPS) were determined again.

At the 12 months recall, periapical radiographs from the experimental teeth were taken. The same experienced periodontist (A.B.) measured the radiographic bone level (BL) at baseline and 12 months as the distance between the CEJ and the most apical level of the alveolar bone. The CEJ was identified on the radiographs as the intersection between the root surface line at the experimental site and the external line of the enamel/restoration. The apical level of the alveolar bone was identified as the intersection between the most apical line of the alveolar bone and the root surface. Radiographic measurements were performed using an internationally used image measuring software (Image J2).

### Statistical analysis

The statistical analysis was performed using a commercially available software program (SPSS for Windows Version 12.0). Mean values and standard deviations were calculated for each variable. The statistical unit was the patient, and the primary outcome variable was CAL gain. Secondary endpoints were mean changes in FMPS, BOP, GBI, PD, PD reduction, CAL-, BS- (ΔBS) and BL-gain (ΔRBL) at 12 months. Statistical significance of intra- and intergroup differences between the two patient groups were determined using non-parametric tests (Mann-Whitney *U* test, Wilcoxon test), while the statistical significance was set at 0.05.

## Results

Thirty patients (mean age 44.38 ± 8.2 years, 11 smokers, 19 women), 15 per treatment group, were included in this RCT. All patients were in the corrective phase of the periodontal treatment (oral hygiene instructions, occlusal adjustments if indicated and non-surgical subgingival debridement had been previously performed, at least 3 months before the corrective surgical phase). None of the patients received adjunctive systemic antibiotics in the initial phase (i.e. adjunctive to non-surgical debridement). All treated teeth in the present study had an adequate static and dynamic occlusion, without interferences (possible interferences in static or dynamic occlusion had been removed at the beginning of the initial therapy).

**Fig. 1:**
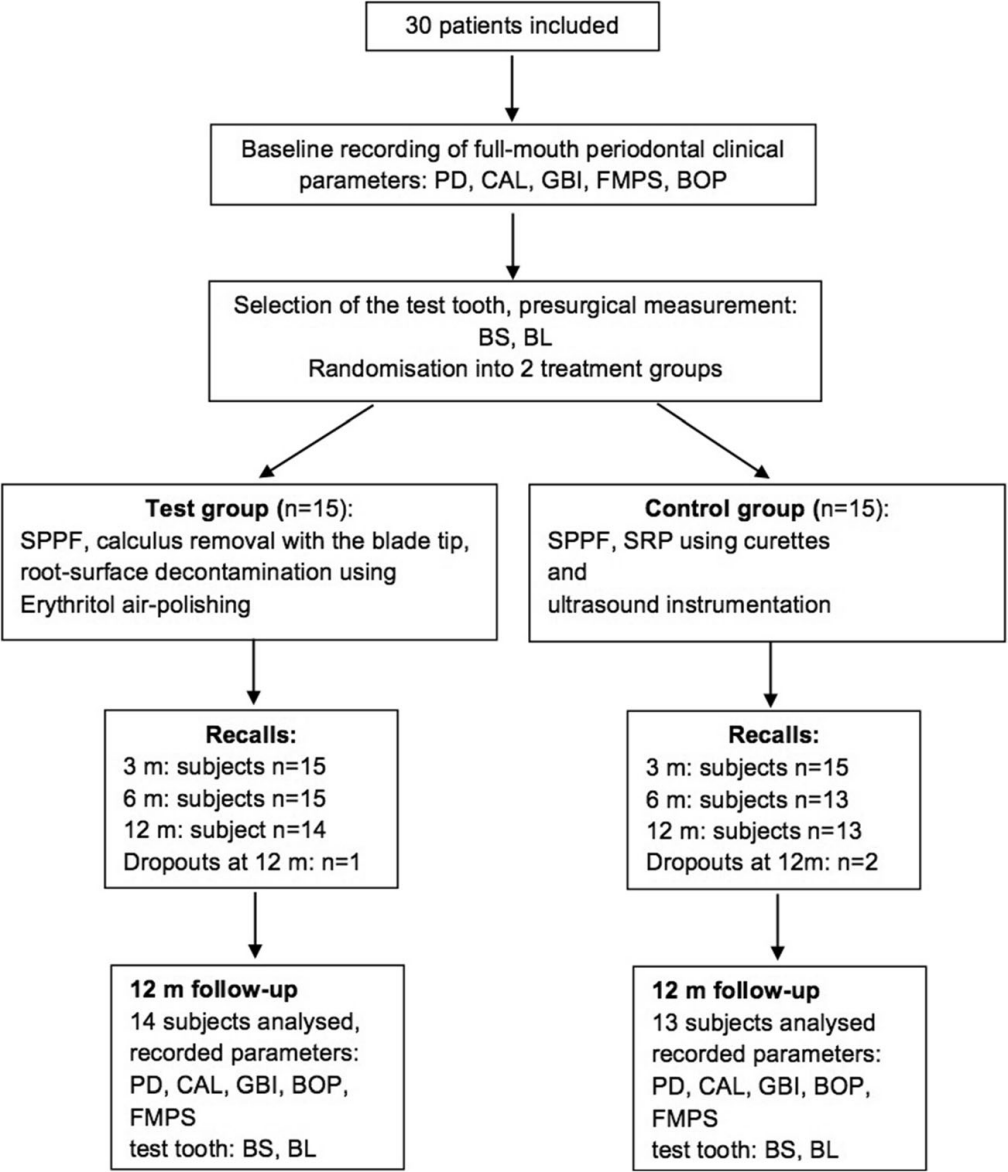
Flowchart of the study. SPPF, simplified papilla preservation flap; PD, pocket depth; CAL, clinical attachment level; BS, bone sounding; BOP, bleeding on probing; GBI, gingival bleeding index; FMPS, full-mouth plaque score; BL, radiographic measurement of the bone level

At the 12-month follow-up, three patients dropped out (reason for drop out: missed the appointment of personal reasons): one in the test and two in the control group (Fig. 1). Demographical data and distribution of the type of experimental teeth are shown in Table 1. Postoperative healing was uneventful in all cases: no oedema, flap dehiscence or allergic reactions were observed.

**Table 1:** Demographics at baseline

Patients	Test *N* = 15	Control *N* = 15
Age (years)	45.4 ± 5.75	44.3 ± 9.71
Female gender (*n*/%)	9/60%	10/66.7
Smokers (*n*/%)	4/26.7%	7/ 46.7%
Test tooth type (*n*/%)		
Incisors	9/60%	12/80%
Canines	3/20%	3/20%
Premolars	3/20%	-

The clinical and radiological results for the treated teeth are shown in Table 2. Six and 12 months after surgery, subjects in both study groups showed statistically significant clinical improvements compared with baseline (*p* < 0.05): lower PD, CAL and BS mean values and statistically significant PD reductions and gain in CAL and BS. At 12 months, BL was also statistically significantly reduced. There were no statistically significant differences between the two treatment groups neither for CAL gain (*p* = 0.469/0.266 for 6/12 months) nor for any of the other evaluated clinical parameters at the two evaluated timepoints (*p* > 0.05, PD, AL, BS). At baseline, however, PD values were statistically significantly lower in the test compared with the control group (*p* = 0.035). At 12 months, a comparable percentage of patients showed “pocket closure” (PD ≤ 3 mm): 78.57% (*n* = 11) in the test group and 76.92% (*n* = 10) in the control group. For all other investigated parameters, no statistically significant differences were detected prior to surgery.

**Table 2:** Clinical parameters at baseline, at 6 m (test: *n* = 14; control: *n* = 13) and 12 months (test: *n* = 14; control: *n* = 13), and their changes (Δ) for the test tooth

Parameters	Test	Control	Test vs. control *p* value
PD base (mm)	6.00 ± 0.00	6.23 ± 0.42	*0.035s*
6 m (mm)	3.00 ± 0.78^S^	2.92 ± 0.86^S^	*0.675*
12 m (mm)	3.07 ± 0.92^S^	2.85 ± 0.99^S^	*0.456*
ΔPD base-6 m (mm)	3.00 ± 0.78	3.35 ± 1.11	*0.309*
ΔPD base-12 m (mm)	3.00 ± 0.96	3.38 ± 1.12	*0.240*
CAL base (mm)	7.87 ± 1.25	8.03 ± 1.54	*0.800*
6 m (mm)	5.86 ± 2.14^S^	5.54 ± 2.15^S^	*0.730*
12 m (mm)	5.43 ± 1.91^S^	5.38 ± 1.94^S^	*0.921*
ΔCAL base-6 m (mm)	2.00 ± 1.80	2.5 ± 1.26	*0.469*
ΔCAL base-12 m (mm)	2.50 ± 1.60	2.85 ± 1.21	*0.266*
BS base (mm)	7.00 ± 0.96	7.27 ± 0.82	*0.222*
6 m (mm)	4.14 ± 0.72^S^	3.88 ± 0.94^S^	*0.422*
12 m (mm)	4.00 ± 0.94^S^	4.04 ± 0.75^S^	*0.916*
ΔBS base-6 m (mm)	2.86 ± 1.17	3.42 ± 1.26	*0.247*
ΔBS base-12 m (mm)	3.11 ± 1.16	3.21 ± 0.75	*0.710*
BL base (mm)	8.06 ± 2.82	7.68 ± 2.01	*0.917*
12 m (mm)	5.91 ± 2.02^S^	6.52 ± 2.41^S^	*0.433*
ΔBL (mm)	2.27 ± 1.52	1.30 ± 1.04	*0.075*

^S^Statistically significant *p* < 0.05

*PD* probing pocket depth, *CAL* clinical attachment level, *BS* bone sounding, *BL* bone level measured on periapical radiographs, *s*: statistically significant

Full-mouth clinical results are shown in Table 3. Patients maintained good oral hygiene (FMPS) and low levels of inflammation (BOP; GBI) without statistically significant changes between baseline and 6 or 12 months respectively or between the two treatment groups (*p* > 0.05) (Table 3).

**Table 3:** Full-mouth clinical parameters at baseline and 12 months

Parameters	Test *N* = 14	Control *N* = 13	Test vs. control *p* value
GBI base (%)	5.57 ± 8.15	4.89 ± 6.85	*0.983*
6 m (%)	5.54 ± 10.05	3.03 ± 7.36	*0.123*
12 m (%)	4.92 ± 6.80	4.13 ± 5.84	*0.666*
FMPS base (%)	23.34 ± 13.14	19.36 ± 13.06	*0.455*
6 m (%)	21.85 ± 9.58	20.26 ± 16.48	*0.299*
12 m (%)	17.57 ± 9.86	23.22 ± 14.08	*0.356*
BOP base (%)	13.06 ± 13.97	11.94 ± 10.78	*1.000*
6 m (%)	11.83 ± 7.32	10.71 ± 6.71	*0.381*
12 m (%)	12.91 ± 12.55	14.21 ± 13.42	*0.685*

^S^Statistically significant *p* < 0.05

*GBI* gingival bleeding index after Ainamo & Bay, *FMPS* full-mouth plaque score after O`Leary, *BOP* bleeding on probing.

## Discussion

The results of the present randomized controlled clinical pilot study indicate that root surface decontamination by means of air polishing using erythritol powder following surgical periodontal therapy in supra-alveolar bony defects results in improvement of the clinical parameters. Additionally, comparable clinical improvements to those obtained with conventional mechanical debridement using hand and ultrasonic instruments in open flap debridement were observed as evidenced by the fact that no statistically significant differences were detectable 12 months postsurgically for all evaluated clinical (PD, CAL, BS) and radiologic (BL) parameters between the two groups (Table 2).

At 6 and 12 months, both treatments led to statistically significant clinical (CAL gain, PD reduction, BS reduction) reductions compared with baseline (*p* < 0.05, Table 2); additionally radiological (BL-reduction) improvements were also measurable at the experimental teeth at 12 months. Despite the fact that the changes in the clinical parameters were slightly lower in the test group compared with the teeth in the control group, no statistically significant differences were detected between the groups (*p* > 0.05). Nonetheless, mean values of baseline PD were statistically significantly lower (*p* = 0.035) in the test compared with the control group. This may rely on the fact that patients were randomized to the treatment according to a block randomization procedure with the limitation that no homogenous patient distribution was assured. However, these differences were low and comparable PD reductions (test: 3.00 ± 0.78/2.93 ± 0.92 mm vs. control: 2.92 ± 0.86 /3.38 ± 1.12 mm), and PD values at 6 and 12 months, respectively (test: 3.00 ± 0.78/ 3.07 ± 0.92 mm vs. control: 2.92 ± 0.86/2.85 ± 0.99 mm), were obtained (Table 2). Moreover, all other evaluated parameters (BS, BL) together with the primary outcome variable (CAL) showed comparable baseline values without any detectable statistical difference between the groups (*p* > 0.05). To the best of our knowledge, at present, no other study has reported so far the use of air polishing for root surface decontamination during periodontal surgery, and therefore, the present results cannot be directly compared.

Nonetheless, two previous clinical studies using comparable study protocols have evaluated the need of root cementum removal during periodontal surgery in supra-alveolar periodontal defects. In line with our results, Nyman et al. [8] showed in 11 patients with 87 test and 85 control teeth (without including molars) and that comparable results for PD and CAL can be obtained 2 years after surgery with either access flap and SRP or with access flap and polishing of the root surfaces with rubber cups without previous mechanical debridement (subgingival calculus if present, had been chipped off with curettes) [8]. Later, Mombelli et al. [6] confirmed the results of Nyman et al. [8]; in seven patients with PD ≥ 6 mm and horizontal bone loss, single-rooted teeth were either mechanically debrided with curettes or not (test teeth) during conventional surgical therapy. At test teeth, visible calculus had been carefully chipped off. They obtained comparable results for all clinical (PD, CAL) and microbiological parameters, without statistically significant differences between the debrided or non-debrided teeth [6]. These clinical results corroborate the present ones, where no differences were detected between the air-polished or the mechanically debrided root surfaces. Moreover, Mombelli et al. [6] emphasized the fact that the alteration of the ecological environment without instrumentation of the root surfaces can lead to periodontal healing with “physiological” PD of 3-4 mm and has a major impact on the subgingival microbiota 12 months postsurgically [6].

The clinical improvements obtained in the control group compare well with those from other control groups of previous studies that have evaluated regenerative surgical therapy in supra-alveolar periodontal defects. Di Tullio et al. [28] obtained in their control group lower values for teeth with horizontal bone loss where the root surfaces of four adjacent teeth had been accessed with a SPPF, scaled and root planed and conditioned with 24% EDTA (CAL gain: 1.04 ± 0.61 mm vs. our study 2.77 ± 1.16 mm; PD reduction: 2.28 ± 0.89 mm vs. our study: 3.38 ± 1.12 mm; BL change: − 0.004 ± 0.79 mm vs. our study 1.30 ± 1.04 mm) [28]. The discrepancy between the results may rely on differences in study protocols: Di Tullio et al. [28] included as experimental sites also sites with PD = 5 mm, whereas in our study, only sites with PD ≥ 6 mm were included. This aspect may have had an impact on the final results since it has been reported that surgical periodontal therapy in shallow sites leads to lower PD reductions and CAL gain as compared with deep sites [8, 29].

In contrast to our study, the teeth in the control group included in the study by Di Tullio et al. [28] were conditioned with 24% EDTA following root debridement and prior to flap closure possibly having an impact on the final outcome. Another difference which may have influenced the results is the inclusion criteria of the patients: a full-mouth bleeding score < 20% accounted for inclusion and only subjects without any periodontal treatment in the previous 2 years had been considered, while in our study, only patients in the corrective phase of the systematic periodontal therapy (min. 3 months after anti-infective therapy) were included. This may have resulted in treating patients with a higher degree of periodontal inflammation in the study of Di Tullio and coworkers than those included in the present RCT (in our study: baseline GBI 4.80 ± 6.85%, baseline BOP 11.94 ± 10.78%; in the study by Di Tullio et al.: no exact value for baseline GBI, only a general remark < 20%, no available BOP value) (Table 3) [28]. An initially higher degree of inflammation may negatively affect the outcomes of periodontal surgery leading to lower CAL gain and PD reductions and greater gingival recessions.

Other authors evaluating the efficiency of enamel matrix derivative in supra-alveolar periodontal defects have included in the control group teeth with PD ≥ 5 mm [29]. These teeth received mechanical debridement after performing a classical access flap (intrasulcular incisions, mucoperiosteal flap). Their results [29] revealed lower mean values in the control group for PD reduction (sites with baseline PD 4–6 mm: 0.61 ± 0.95 mm, PD ≥ 7 mm: 1.95 ± 1.17 mm) and CAL gain (sites with baseline PD 4–6 mm: 0.36 ± 0.78 mm, PD ≥ 7 mm: 0.78 ± 0.62 mm) as opposed to the present study (ΔPD: 3.38 ± 1.12 mm, ΔCAL: 2.77 ± 1.16 mm). Similarly, lower CAL gain and PD reductions were obtained by Yilmaz et al. [30] (ΔPD: 1.53 mm, ΔCAL: 0.54 mm). Nonetheless, different types of access flaps had been used in these studies: in Jentsch and Purschwitz [29] conventional access flap with intracrevicular incisions and in Yilmaz et al. [30] intracrevicular and reverse bevelled incisions. In the present study, SPPS had been used in order to perform a more minimally invasive approach with minimal bone resorption and gingival recession after flap elevation. It has been repeatedly demonstrated that SPPF represents more minimally invasive surgical procedure by preserving the interproximal vascular plexus and diminishing the microvascular damage, sustaining a faster organization of the granulation tissue [31]. This corroborates the data obtained in intrabony defect and supports the added benefit of using SPPF over conventional surgical techniques [32–34]. However, it has to be kept in mind that in all of the abovementioned studies [28–30], multi-rooted teeth (i.e. molars) have been also included, while in the present study, only single-rooted teeth have been considered. It has been repeatedly demonstrated that clinical results obtained following surgical periodontal therapy at multi-rooted teeth (i.e. molars) are generally poorer than those obtained at single-rooted teeth, due to the presence of furcation defects or concavities which may represent bacterial niches more difficult to access for cleaning [35–38]. Based on this fact, in order to diminish any other factors that may interfere with the investigated surgical protocols, we have included in the present study only single-rooted teeth using for surgical access the SPPF. All treated teeth were located in aesthetically demanding areas, where a conventional resective therapy with osseous recontouring may have had a negative impact on the aesthetic outcomes.

Limitations of the present study include the fact that the baseline PD values were statistically significant different in the two study groups possibly due to the used type of randomization (block randomization). This type of randomization assures a balance in the sample size; however, differences between the groups regarding certain covariates may be seen. Nonetheless, it is important to stress at this point out that this study was designed as a feasibility study in order to primarily assess the possibility of using air-polishing devices as a decontamination method during periodontal surgery in order to use a more minimally invasive decontamination procedure compared with the standard mechanical debridement with hand and ultrasonic devices. Thus, for future studies, well-planned RCTs with a stratified type of randomization that assures an equal baseline distribution of the patients with regard to smoking, age and test sites should be considered. A further study limitation is the fact that no individualized acrylic stents for performing the radiographs had been used, and therefore, the present outcomes in terms of BL changes have to be interpreted with caution in the light of the clinical outcomes. However, this was not the aim of the study just a secondary observation. Considering that dropouts from the study could not be prevented, the power of the study has been recalculated for the main outcome variable (CAL gain); thus, a power of 91% has been obtained for an α = 0.05 (post hoc analysis).

Taking into consideration that the study was designed as a randomized controlled clinical pilot study, we did not intend to state and prove a certain non-inferiority/superiority hypothesis. Thus, based on the current findings, future studies may be planned in order to test the non-inferiority of air polishing during periodontal flap surgery as compared with conventional mechanical root surface debridement.

Taken together, the present results together with the available data from preclinical and clinical studies suggest that air polishing with erythritol is an efficient for biofilm removal assuring less substance loss compared with mechanical debridement with hand and ultrasonic instruments [23].

Within their limits, the present results indicate that the use of air polishing with an erythritol powder during periodontal surgery may represent a valuable minimally invasive adjunct following calculus removal by means of hand and ultrasonic instruments or a valuable alternative to these, for root surfaces without calculus.

Future well-designed RCTs with sufficient power should be considered in order to clearly define the comparability of these two debridement procedures during periodontal surgery.
